# Effects of a person-centered music-based intervention in the rehabilitation of older adults with mild to moderate dementia

**DOI:** 10.1177/25424823251367291

**Published:** 2025-09-15

**Authors:** Sara Santini, Alessandra Merizzi, Maria Joao Azevedo, Sandra Costa, Ioana Caciula, Mirko Di Rosa, Sabrina Quattrini

**Affiliations:** 1 Center for Socio-Economic Research On Aging, 9345 IRCCS INRCA-National Institute of Health and Science On Aging, Ancona, Italy; 2 Associação - Sons do Estaminé, Trofa, Portugal; 3 Asociatia Habilitas - Centru de Resurse Si Formare Profesionala, Bucharest, Romania; 4 Center for Biostatistic and Applied Geriatric Clinical Epidemiology, 9345 IRCCS INRCA-National Institute of Health and Science on Ageing, Ancona, Italy

**Keywords:** Alzheimer's disease, dementia, music, non-pharmacological intervention

## Abstract

**Background:**

With the progressive population aging, dementia is reaching epidemic dimensions worldwide. Non-pharmacological music-based interventions can have a positive impact on the rehabilitation of older adults with dementia. Nevertheless, there are few longitudinal cross-national studies testing their impact.

**Objectives:**

This pilot study aims at shedding light on the effects of the SOUND person-centered music-based intervention on well-being, cognition, executive functions and mood of older adults with mild-moderate dementia in three European countries.

**Methods:**

An original intervention consisting in 12 sessions of active and passive music activities (singing, rhythmic exercises with Orff's tools, narratives elicited by music, etc.), led by a trained facilitator, was implemented in Italy, Portugal and Romania with 41 older adults with mild-moderate dementia attending elder facilities. Data on well-being, cognition, executive functions and mood of participants were collected before, at the end and two weeks after the intervention through psychometric tools. Temporal comparisons were assessed by T-test for paired samples.

**Results:**

The SOUND intervention significantly improved participants’ well-being, cognition and executive functions over time and remained stable at the follow-up at cross-national level. The potential of the intervention on mood is not clear due to depression and anxiety increasing among Romanian participants.

**Conclusions:**

Cross-national, longitudinal, multidisciplinary mixed-method studies demonstrating the effects of music-based rehabilitative interventions for older adults with mild-moderate dementia are encouraged to shape innovative treatments as well as to identify possible adverse effects on participants’ mood linked to scarcity of coping capabilities as source of distress in older individuals.

## Introduction

Over 55 million people live with dementia worldwide,^1^ and this number is expected to triple by 2050 considering the increase of population longevity and the fact that aging is one of the main factors associated with dementia.

Currently, only a few drugs are specifically authorized for reducing dementia's deterioration, intended exclusively for Alzheimer's disease (AD). These medications allow symptoms to be controlled for some time, but they cannot stop the progression of the disease plus not all patients respond to such treatments.^2,3^ Therefore, the combination of drug therapy and psychosocial non-pharmacological interventions seems to be promising in the treatment of dementia, having positive effects on neuropsychiatric symptoms, depression, and agitation.^4^

Numerous psychosocial interventions for dementia are grounded in Kitwood's Person-Centered Care approach, that prioritizes the person over the evidence and technicalities of interventions by emphasizing the meaning of relationships and communication when meeting the individual's needs,^5^ and facilitates the inbuilt actualizing tendencies of individuals.^6^ Five core psychological needs of people with dementia were identified by Kitwood,^7^ i.e., attachment, inclusion, occupation, identity and comfort, all of them linked to the central need for love. Kitwood also identified 17 negative interactions (detractors) that diminishes personhood and grouped them under the term “malignant social psychology” (invalidation, infantilization, intimidation, labeling, stigmatization, withholding, accusation, outpacing, treachery, disempowerment, disparagement, objectification, ignoring, disruption, banishment, imposition, and mockery) and 12 positive interactions that empower dignity and personhood, named as “positive person work” (recognition, holding, play, collaboration, negotiation, facilitation, celebration, creation, timalation, giving, and relaxation).^7^ Music-based care interventions seem to be well-aligned with Kitwood's model because of the natural capability of music to contribute to building personhood, improving quality of life, enhancing communications,^8–10^ and eliciting emotions and feelings, which are among the essential aspects of person-centered dementia care.^11^

Music is considered a great resource to stimulate the brain, thanks to the powerful and various sensory, cognitive, emotional, and motor experiences it can elicit,^12^ and to its capability of boosting neuroplasticity.^13^ In fact, the neural networks involved in music processing connect the temporal, frontal and parietal cortex with the limbic areas and with the cerebellum, triggering neuroplasticity.^14^ Thus, music can provide a comprehensive, neurological scaffold for memory and cognition.^15^

Since music can evoke autobiographical and emotional memories, processed in the anterior hippocampus,^16,17^ people with AD can maintain intact the ability to recognize familiar melodies, and even in the advanced stage of the disease, the ability to sing along with music.^16^

Music has been proved to have beneficial effects on pleasure, reward, arousal, stress, motivation, immunity and social affiliation.^18^ Moreover, Toader et al.^19^ highlighted the multiple neuropsychological mechanisms of music processing in neurological rehabilitation, including mirror neurons, neuroplasticity and neural synchronization, that translate into psychosocial, cognitive, behavioral, and motor improved outcomes.

Music therapy, along with other non-pharmacological approaches based on music, may include a variety of models and techniques, ranging from musical improvisation, receptive music listening, composition, music and images, singing, musical performance, learning through music, music-assisted relaxation, adapted music intervention, and movement to music.^20^ In the last ten years several studies have shown the positive effects of music-based interventions on different realms of people with dementia: such interventions can improve cognition,^15,21–23^ and sense of self and identity,^24^ relieve depression or anxiety,^25–27^ reduce neuropsychiatric symptoms and therefore improve (social) behavior.^24,28–30^

While many studies show the effects of music-based intervention on cognition and behavior of older adults with dementia (OAD), as reported above, few literatures show the impact of music on general well-being,^26,28,31–33^ topic that remains a partly under-covered by research.

Although the cited studies show positive results of music-based interventions with OAD and they encourage their use, Van Der Steen points out that there is no evidence of their effects on agitation and cognition and on anxiety,^28^ especially in the long-term, albeit they may improve social behavior,^29^ and Moreno-Morales highlights the need to shed light on the effects of such interventions on cognition and mood in the short and long-term, through longitudinal studies.^34^ Moreover, international studies on this topic are rather rare (e.g., the MIDDEL study),^35–37^ probably due to the fragility of the target, that is difficult to engage, the different types of professionals involved (e.g., music therapists, neurologists, musicians, psychologists, gerontologists, dementia care professionals), the heterogeneity in the interventions and assessment methodologies, and the economic resources needed.

This pilot study aims at contributing to the scientific debate and covering the literature gap by shedding light on the short and mid-term effects of an intervention conducted within the SOUND study (“Training Social and health care prOfessionals in mUsic-based therapeutic iNterventions to support older people with Dementia”, funded by the Erasmus + Programme, https://soundeuproject.eu/), on cognitive and executive functions, mood and well-being of 41 older adults with mild-moderate dementia (OAMD) living in Italy, Portugal and Romania.

## Methods

### Study design

A quasi-experimental mixed-methods study protocol was carried out to assess the efficacy of an original music-based intervention on well-being, cognition, executive functions and mood of OAMD in view of a full-scale study.^38^

This pilot study is aimed at answering the following research question: To what extent can the SOUND intervention improve cognitive and executive functions, mood and well-being of older adults with mild-moderate dementia?

The impact of the SOUND intervention on OAMD was assessed in three different moments, at the baseline (T0), at the end of the intervention (T1) and two weeks after (T2), through a questionnaire including psychometric scales (described in paragraph 2.6) and live monitoring observational tools. In this paper, only the outcomes of the analysis of quantitative data are reported due to the richness of qualitative ones requiring a dedicated paper. The SOUND intervention, framed in the Kitwood's person-centered approach,^39^ was implemented between October 2023 and February 2024 in Italy, Portugal and Romania.

### Sampling: Inclusion criteria and recruitment strategies

The study design planned to involve 45 OAMD in total (15 per country), through a sampling for meaning procedure.^40^ In Italy, participants have been recruited among the guests of the Alzheimer day-care center at the National Institute of Health and Science on Aging (INRCA). In Portugal, they were selected among residents of three care facilities at Santa Casa da Misericórdia de Santo Tirso and the community center Centro Comunitário da Trofa - ASAS. In Romania they were selected among the guests of two care facilities in Bucharest.

OAMD were included in the experimentation if they were 65 years of age or older; showed interest in the project; were able to see, hear and move (even with aids); were diagnosed with mild to moderate cognitive impairment according to the Montreal Cognitive Assessment (MoCA),^41^ and scored ≥10/30. Moreover, they did have not to experience aphasia (mild acceptable) and did have to be able to understand and undertake simple tasks as required during activities.^38^

Participants were screened two weeks before the beginning of the intervention by psychologists who administered the MoCA test face-to-face. Depression/anxiety were not an inclusion/exclusion criterion, because, given the scarce evidence of the effect of music intervention on these dimensions,^28^ and the piloting nature of the study, the researchers were interested in understanding how they might change over the treatment, regardless of the initial scores.

### Ethical issues

The cross-national study was approved by the IRCCS INRCA Ethic Committee on 26 May 2023 with General Director Communication number 234 provided on 9 June 2023. In Portugal, the study design was also approved by the Ethical Commission of the CICS - Centro Interdisciplinar em Ciências da Saúde - ISAVE (Instituto Superior de Saúde, Portugal) on 6 December 2022. In Romania, the Ethic Committee approval was not mandatory since the research had already been approved by the IRCCS INRCA Ethic Committee as a cross-national study.

Informed consent was given to the older participants and their relatives who played the role of main caregiver, and they were asked to sign it. The selected individuals were enrolled in the intervention only after signing the informed consent.

### The music intervention

#### A person-centered intervention

The SOUND intervention was framed in the “person-centered” Kitwood's model.^39^ In line with Kitwood's positive person work, SOUND was designed to value each OAMD as a unique individual, and consider their perspective by collecting their biography, respecting their preferences, fears, weaknesses, strengths, and talents, and thus highlighting individuals’ residual capabilities over the lost ones. Thus, the intervention was designed to stimulate positive interactions like holding and collaboration by promoting and enhancing the interpersonal relationships of participants with peers and dementia care professionals (DCPs) (e.g., nurses, professional educators, psychologists, and logopedists), by means of music activities made in group and in circle, and by providing a safe environment where the individuals could express their psychological needs (attachment, comfort, identity, inclusion, occupation and love) by singing, moving, playing instruments and creating narratives under the music elicitation. SOUND also emphasizes communication and meaningful relationships and prioritizes the person's well-being. The musical activities were game-based and assumed full cooperation between OAMD and between the latter and the dementia care professionals. The musical exercises were designed to stimulate the creativity and based on improvisation, as participants could respond to the facilitator's initial stimuli in a personal way, even giving a new direction to the activity. Each response was welcomed to reinforce the participants’ sense of efficacy and avoid any form of judgement.

Finally, SOUND centralizes the person with dementia by making the physical environment esthetically appealing, quiet, warm and welcoming, e.g., by a) preparing the chairs with the persons’ name and disposing participants near other OAMD and DCPs they had a good relationship with; b) playing a soft welcoming music while they were entering in the room; c) greeting each one by name.

#### Structure, activities, and team

The intervention included 12 sessions delivered twice a week, for six consecutive weeks. Each session lasted about 45 min and was divided into four different phases: (1) welcoming; (2) opening activity; (3) three to five main activities depending on the length and intensity of each one; (4) closing activity. During every phase, except for phase 3, one exercise was carried out.

The intervention included both active (vocal and rhythmic production) and passive (listening) music-based activities. Additionally, facilitators proposed narrative activities elicited by music and free association with it, such as creating or telling stories, talking about pictures, describing an object or a feeling and so on. All activities had the general objective of enhancing participants’ wellbeing and a secondary objective to stimulate specific cognitive abilities.

Each activity was chosen based on (a) participants’ personal music preferences and life-story, identified by means of a biographical sheet; (b) participants’ level of physical, cognitive and mental health condition; (c) the objective of cognitive stimulation, e.g., verbal fluency, memory, coordination. For example, since in one group there was a person who loved dancing, the session included one activity based on simple dance movements and another for which a dancing tune was played.

Activities were also organized by considering particular participants’ fears and triggering factors based on their personal stories and current situations. Thus, the teams changed the planned activities time to time in accordance with the special condition of the participants.

The music tracks were chosen also by considering the musical traditions at the country level, thus including singers belonging to the popular music culture such as Domenico Modugno in Italy, Amália Rodrigues in Portugal and Constantin Drăghici in Romania.

The team that delivered the intervention included: a facilitator, and internal and external observers. All were previously trained to the SOUND method.^42^

The facilitator proposed the activities to the participants in a responsorial style, led the circle, welcomed and responded to any spontaneous and unexpected activities coming from the participants and proposed them back to the whole circle.

The activities were led by five female facilitators (one in Romania and two in Italy and in Portugal). In Romania, the sole facilitator in both groups was a music and piano teacher. In Italy, the two facilitators were: (1) an occupational therapist working at the dementia day-center with a music background, and (2) a lyric singer and teacher. In Portugal, both facilitators were educators (one with music background) in the institution hosting the intervention.

The internal observers were DCPs who had the task of supporting older participants during the intervention in a non-intrusive and facilitating way. In the circle, there was a DCP every two OAMD. They took part in the activities and notified the facilitator of any signal of discomfort from the nearest older adults.

Four researchers (i.e., two psychologists and two gerontologists in Italy; two psychologists, one social worker, one professional in the field of aging in Romania; one psychologist, two musicians and one social educator in Portugal) sat outside the circle (for covering all the sections of the circle circumference) and acted as external observers of participants’ emotional and behavioral responses to the proposed activities during each session. They used an observational tool and took notes which were then transcribed in a dedicated diary for each participant.

In Italy and Romania, OAMD were divided into two groups, made respectively of seven and eight persons, assisted by the same number of care professionals. In Portugal the first group was made of ten OAMD and eight DCPs, while the second group included eight OAMD and seven DCPs. To cover participants’ absence, some DCP rotated in the two groups to assure the number of internal observers was sufficient in comparison to the OAMD attending the sessions.

### Outcome variables and research tools

Table 1 shows an overview of the outcome variables and research tools. An exhaustive description of the research tools has been published on a scientific paper describing the study protocol.^38^ Here we limit to list the outcome variables and recall the measuring tools (Table 1). OAMD's cognition was measured through the MoCA,^41^ and the Frontal Assessment Battery (FAB).^43^ Mood (depression and anxiety) through the Hospital Anxiety and Depression Scale (HADS).^44^ Well-being through the WHO Wellbeing Index (WHO-5).^45^

**Table 1.** Outcome variables and research tools.

Outcome variables	Research tool
Well-being (primary outcome)	a. WHO-5 (WHO (Five) Well-Being Index)
Cognition (general cognition + executive functions)	MoCA (Montreal Cognitive Assessment)
	FAB (Frontal Assessment Battery)
Mood (anxiety and depression)	HADS (Hospital and Anxiety Depression Scale)

### Statistical analysis

The quantitative data analysis included outcomes description. After having verified normality in distribution of continuous variables via Shapiro-Wilk test, mean and standard deviation were reported and differences across Countries (Italy, Romania, and Portugal) were evaluated via one-way analysis of variance (ANOVA).

Absolute frequency and percentage were reported for categorical variables and differences among Counties were assessed using Chi Squared test. To visualize the average trend of the outcome variables for three Countries, predicted values (fitted lines) and 95% confidence intervals (95%CI) were plotted using two-way graphics and statistical significance was assessed using repeated measures ANOVA. Temporal comparisons (T0 versus T1, T0 versus T2, or T1 versus T2) were additionally assessed by T-test for paired samples. Subjects withdrawing from the study were not replaced, according to the intention to treat (ITT) principle. Statistical analyses were conducted by a statistician, who was blind to group allocation prior to analysis. The significance threshold was always set at *p* < 0.05. The software used for the analyses was StataNow/MP 18.5. Sequential imputation using chained equations method was applied in case of missing values in covariates.

## Results

### Participants’ characteristics

In the three countries, 48 OAMD were recruited (15 in Italy and in Romania and 18 in Portugal), but only 41 were considered in the analysis: seven participants did not attend at least six out of 12 sessions, or they could not be assessed during T1-T2 due to poor health condition. Thus, the final sample is made of 13 OAMD from Italy, 13 from Portugal and 15 from Romania.

Table 2 provides details about the most relevant socio-demographic characteristics of the participants, where the statistically significant differences (*p*) among pilot sites are written in bold. OAMD were diagnosed with dementia/cognitive impairment for 3.4 years on average, with a standard deviation of 2.2 years, indicating variations in the duration of the disease among participants.

**Table 2.** Participants’ characteristics.

	Italy	Romania	Portugal	Total	*p*
	N = 13	N = 15	N = 13	N = 41	
Age (mean years ± SD)	81.7 ± 8	80.4 ± 9.1	79.8 ± 9.3	81.0 ± 3.4	0.781
Years from dementia diagnosis (mean years ± SD)	3.8 ± 2.2	3.3 ± 2.2	3.1 ± 2.5	3.4 ± 2.2	0.751
Sex, N (%)					0.218
male	6 (46.2)	4 (26.7)	2 (15.4)	12 (29.3)	
female	7 (53.8)	11 (73.3)	11 (84.6)	29 (70.7)	
Years in education, N (%)					**0.013**
0–8	7 (53.8)	9 (60)	13 (100)	29 (70.7)	
9–12	3 (23.1)	5 (33.1)	0 (0)	8 (19.5)	
13+	3 (23.1)	1 (6.7)	0 (0)	4 (9.8)	
Place of living, N (%)					**<0.001**
home	13 (100)	0 (0)	2 (15.4)	15 (36.6)	
residential care	0 (0)	15 (100)	11 (84.6)	26 (63.4)	
Living with, N (%)					**<0.001**
alone	3 (23.1)	0 (0)	0 (0)	3 (7.3)	
spouse/partner	8 (61.5)	0 (0)	2 (15.4)	10 (24.4)	
Others (children, in laws, grandchildren)	4 (30.8)	0 (0)	1 (7.7)	5 (12.2)	
residential care	0 (0)	15 (100)	11 (84.6)	26 (63.4)	
Language comprehension and oral expression, N (%)					0.545
Low	1 (7.7)	2 (13.3)	1 (7.7)	4 (9.8)	
Medium	10 (76.9)	7 (46.7)	7 (53.8)	24 (58.5)	
High	2 (15.4)	6 (40)	5 (38.5)	13 (31.7)	

Participants’ average age was 81.0 years (SD = 3.4) with an overall majority of women (70.7%), mostly in Portugal (84.6%) and less in Italy (53.8%). Within the overall sample, more than two thirds of OAMD were low educated (70.7%); the most educated OAMD were in Italy with 23.1% that had over 13 years of education corresponding to at least a high school degree.

At cross-national level, 63.4% OAMD were institutionalized. 100% Romanian and 86.4% Portuguese participants lived in residential care, while all Italian participants lived at home mainly with their spouse/partner (61.5%) and attended the day-care center.

At cross-national level, the difference in participants’ educational level, place of living and living conditions was statistically significant.

Language comprehension and oral expression were assessed by interviewing informal caregivers (where available); 31.7% had a high level of both abilities, higher among Romanian and Portuguese participants, while 58.5% had an average level, and 9.8% had a low level of these skills.

### Outcomes description

Table 3 shows the effect of the intervention on the participants well-being, cognition and mood in the three national samples and in the overall sample overtime, i.e., comparing T0 and T1, T1 and T2, and T0 and T2. The statistically significant differences in the three data collection waves are written in bold.

**Table 3.** Effects of the intervention on the outcome variables over time.

Outcomes	T0		T1		T2		*p* (T0-T1)	*p* (T1-T2)	*p* (T0-T2)
	N (%)	mean ± SD	N (%)	mean ± SD	N (%)	mean ± SD			
Well-being									
Italy	13 (100)	54.15 ± 17.41	13 (100)	80.92 ± 13.28	13 (100)	78.15 ± 11.39	**0**.**000**	0.406	**0**.**000**
Romania	15 (100)	65.87 ± 25.56	15 (100)	65.07 ± 25.81	15 (100)	65.07 ± 25.81	0.915	1.000	0.915
Portugal	13 (100)	59.08 ± 17.45	13 (100)	65.54 ± 18.66	13 (100)	66.46 ± 29.15	0.211	0.861	0.222
Total	41 (100)	60.00 ± 20.86	41 (100)	70.24 ± 21.09	41 (100)	69.66 ± 23.70	**0**.**010**	0.758	**0**.**015**
Cognitive functions									
Italy	13 (100)	13.38 ± 2.90	13 (100)	14.38 ± 4.31	13 (100)	13.38 ± 2.90	0.193	**0**.**048**	1.000
Romania	15 (100)	11.07 ± 3.77	15 (100)	14.80 ± 5.29	15 (100)	15.33 ± 5.52	**0**.**001**	0.664	**0**.**007**
Portugal	13 (100)	13.38 ± 3.25	13 (100)	15.00 ± 5.29	13 (100)	15.08 ± 4.03	0.258	0.945	0.154
Total	41 (100)	12.54 ± 3.46	41 (100)	14.73 ± 4.49	41 (100)	14.63 ± 4.76	**0**.**001**	0.866	**0**.**005**
Executive functions									
Italy	13 (100)	10.62 ± 3.18	13 (100)	11.62 ± 3.45	13 (100)	11.00 ± 3.32	0.139	0.312	0.579
Romania	15 (100)	10.67 ± 3.56	15 (100)	12.60 ± 2.59	15 (100)	12.87 ± 3.11	0.077	0.390	0.062
Portugal	13 (100)	8.77 ± 3.17	13 (100)	10.62 ± 8.65	13 (100)	11.62 ± 2.99	**0**.**008**	0.097	**0**.**003**
Total	41 (100)	10.05 ± 3.35	41 (100)	11.66 ± 3.13	41 (100)	11.88 ± 3.16	**0**.**001**	0.452	**0**.**001**
Depression									
Italy	13 (100)	3.92 ± 3.28	13 (100)	2.46 ± 1.56	13 (100)	1.85 ± 2.27	**0**.**030**	0.380	0.058
Romania	15 (100)	3.60 ± 3.20	15 (100)	6.20 ± 2.30	15 (100)	6.20 ± 2.88	**0**.**012**	1.000	**0**.**008**
Portugal	13 (100)	3.15 ± 2.88	13 (100)	2.31 ± 3.77	13 (100)	3.62 ± 5.27	0.271	0.374	0.706
Total	41 (100)	3.56 ± 3.07	41 (100)	3.78 ± 3.50	41 (100)	4.00 ± 4.02	0.676	0.666	0.497
Anxiety									
Italy	13 (100)	4.23 ± 3.49	13 (100)	1.85 ± 1.91	13 (100)	0.77 ± 0.93	**0**.**008**	**0**.**020**	**0**.**002**
Romania	15 (100)	5.47 ± 4.21	15 (100)	8.33 ± 3.83	15 (100)	8.33 ± 4.15	**0**.**018**	1.000	**0**.**018**
Portugal	13 (100)	3.77 ± 4.15	13 (100)	1.46 ± 2.57	13 (100)	1.38 ± 2.63	**0**.**028**	0.776	**0**.**006**
Total	41 (100)	4.54 ± 3.94	41 (100)	4.10 ± 4.34	41 (100)	3.73 ± 4.58	0.511	0.050	0.243

In the overall sample and in the Italian one, the effects of the SOUND intervention on well-being are evident, while no significant effect is observed in the other two countries taken separately. Considering the overall results, the well-being of OAMD significantly increased from T0 (M = 60.0) to T1 (M = 70.24) and then it slightly decreased two weeks after the end of the intervention (T2) but still remains higher than the initial level (M = 69.66). The difference between the baseline and the end of the intervention is statistically significant (*p* = 0.010) as well as the difference between the end of the intervention and the follow-up (*p* = 0.015).

Concerning cognition, the total average MoCA's score increased significantly during the SOUND intervention, from T0 (M = 12.54) to T1 (M = 14.73), with a statistically significant difference (*p* = 0.001), and remained almost stable until T2 (M = 14.63). The significant increase in the cognitive capabilities is confirmed also between T0 and T2 (*p* = 0.005). This suggests a significant and sustained improvement in participants’ cognition as a result of the intervention. At a national level, a similar trend is observed only in Romania, with the difference that a further increase in cognitive capabilities is also observed between T1 and T2, although not with a significant difference. In Portugal, despite a slight improvement, no statistically significant difference is observed in the cognitive capabilities between the three observation points. In Italy, the raise of the cognitive capabilities was not statistically significant between T0 (M = 13.38) and T1 (M = 14.38), and the score went back to the initial level as soon as the intervention ended, i.e., in T2 (M = 13.38).

In the overall sample, at a cross-national level, the executive functions (i.e., conceptualization, mental flexibility, programming, sensitivity to interference, inhibitory control, environmental autonomy as measured through the FAB) significantly increased both from T0 to T1 (M = 10.05 versus M = 11.66), and from T0 and T2 (M = 11.88) with a stable *p* value (0.001). This trend is confirmed in the Portuguese subsample where the improvement is statistically significant (T0-T1 *p* = 0.008 and T0-T2 *p* = 0.003), while in the Romanian and in the Italian subsamples the improvement is not statistically significant despite the positive trend.

The participants’ mood, i.e., anxiety and depression, was measured by the HADS scale. Concerning the level of depression, in the overall sample no significant differences were observed at the three observation points, probably due to the polarized data observed in Romania between T0 (M = 3.60) and T1 (M = 6.20) and T2 (M = 6.20), compared with the other two countries. While in Portugal there is a slight oscillation of the score (not statistically significant), in Italy depression symptoms significantly decreased from T0 (M = 3.92) to T1 (M = 2.46) (*p* = 0.030) till the end of the intervention and then it continued to decrease even if not significantly (*p* = 0.058). Noteworthy, in Romania the trend was the opposite: average depression scores almost doubled from T0 (M = 3.60) to T1 (M = 6.20) with a statistically significant difference (*p* = 0.012) and the score was maintained at T2 (*p* = 0.008).

Similarly, anxiety symptoms decreased during the intervention both in Italy (from M = 4.23 in T0 to M = 0.77 in T2) and in Portugal (from M = 3.77 in T0 to M = 1.38 in T2) and it continued to significantly decrease till the follow up in both countries. On the contrary, in Romania the average anxiety scores increased from M = 5.47 to M = 8.33 between T0 and T1 (*p* = 0.018) and remained stable at T2 (*p* = 0.018 compared to T0).

**Figure 1.**
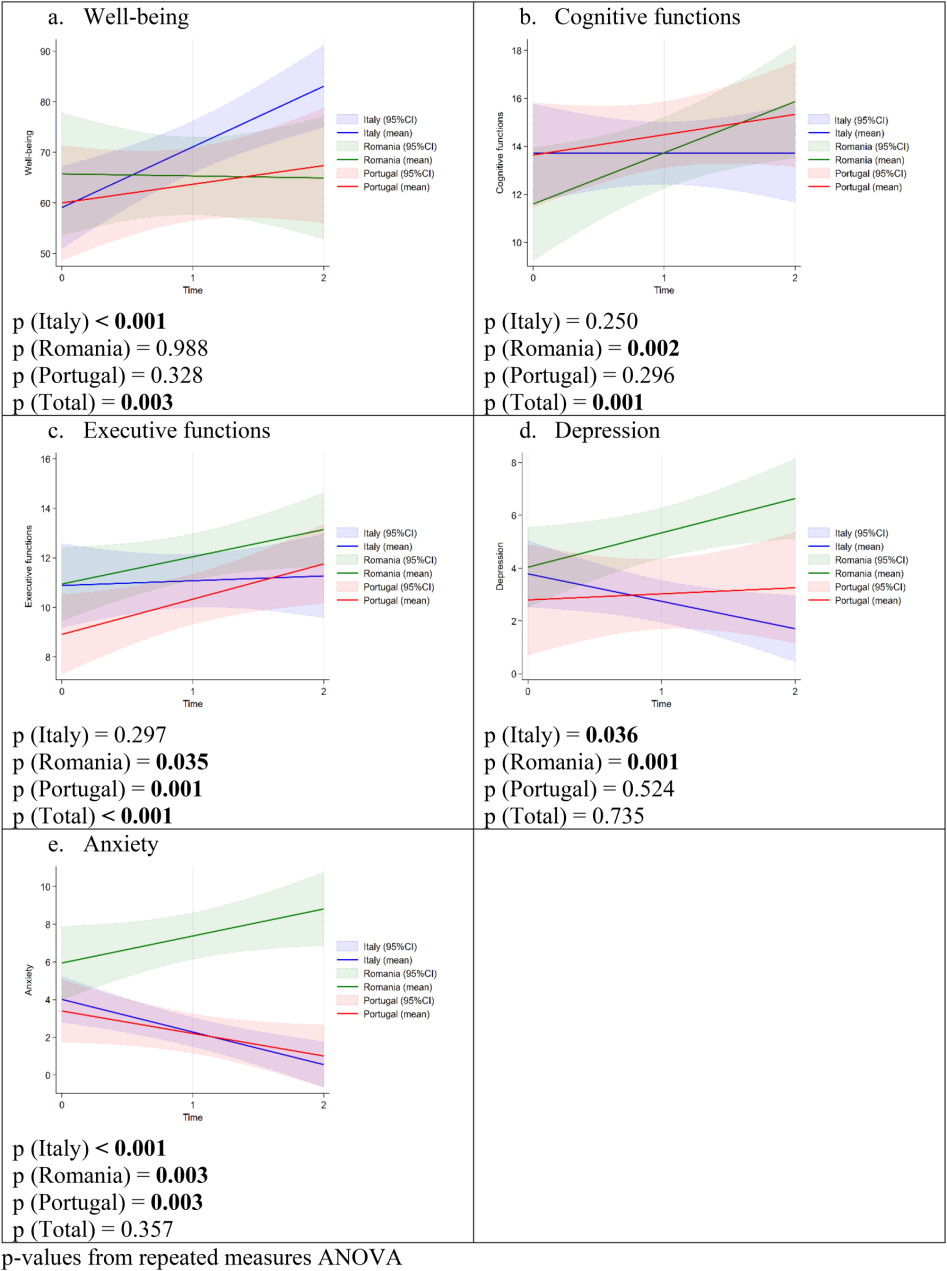
Average trend of the outcomes variables for three countries, fitted lines (95%CI). *p*-values from repeated measures ANOVA. ANOVA: analysis of variance.

Figure 1 reports the average trend of the outcomes in the three national samples and in the overall sample overtime. For well-being (Figure 1(a)) it can be observed that the positive trend was statistically significant only for Italy, while in the case of cognitive functions (Figure 1(b)) the growing trend is significant only for Romania. As regards executive functions (Figure 1(c)), increasing fitted lines were statistically significant for both Romania and Portugal. HADS outcomes (Figure 1(d) and (e)), instead, had contrasting trends across Countries. Romania showed an overtime increment for both depression and anxiety in a statistically significant way; on the contrary, Italy reported decreasing trends for these outcomes, in both cases with *p* < 0.05; Portugal, finally, showed almost no change for depression and a statistically significant decline for anxiety.

## Discussion

### Study outcomes interpretation

SOUND intervention is one of the few longitudinal cross-national studies monitoring the effectiveness of music-based non-pharmacological interventions on older adults with mild to moderate dementia (OAMDs), and one of the few studies focusing on the impact of such interventions on their well-being in addition to cognition and mood.

The study added knowledge to the available literature of OAMD by highlighting the positive effect of the intervention on participants’ well-being in the short,^46^ as well as in the long-term,^26,28–33^ and on general cognition and executive functions.^21–23,25^

The study cannot confirm nor disconfirm the results of the previous literature on the capability of music activities to decrease OAMD's level of depression and anxiety,^25–27^ due to the lack of consistency between participants’ level of depression in Romania compared to the other two countries.

Previous studies indicate that non-pharmacological rehabilitative interventions may also have adverse effects in older adults with cognitive deficits,^47^ also in the long-term because inadequate coping capabilities to the new stimuli, can trigger dyshomeostasis and lead to frustration and distress, due to age-related alterations in hypothalamic-pituitary-adrenal axis.^48^ Thus, Romanian older adults, might have experienced distress that led to emotional decline, due to elevated cortisol and inflammation levels.^49^ This may have occurred among the residents of the Romanian (and not the Portuguese) nursing home due to the poor development of the LTC system in this country,^50^ and the low quality of the assistance in elder care facilities.^51^ Thus, under unfavorable social and relational conditions, musical activities represented an additional source of stress for the older adults.

### Study limitations

The study has limitations, some inherent in any pilot study protocol. The first one concerns the small (seven dropouts further deplete the sample power) and purposive sample size that cannot allow the generalization of the results to a wider population of older adults with mild to moderate dementia. The smallness of the sample did not permit a comparison of the outcomes by participants’ living condition (i.e., older adults attending day-care center versus older adults living in residential care), because this further division of the sample would have detracted from the analysis power. It also dissuaded researchers from correcting the T-test by multiple comparisons.

Second, the lack of a control group, not foreseen in the pilot studies, limited the identification of exogenous factors influencing the impact of the intervention on the outcome variables. Noteworthy, the researchers could not properly take under control factors such as care and relational situations or meaningful events (e.g., going back home from the facility for Christmas holiday altering the mood of some OAMD) that might have influenced the well-being, cognition and mood of OAMD outside of the intervention. Similarly, it was not possible to assess to what extent the improvement in participants’ well-being, cognition and executive functions depends on musical rather than on communal group activities.

Moreover, the short duration of the intervention might have penalized its effectiveness. In fact, despite there is no consensus in the literature regarding the minimum number of sessions and the frequency required for music-based interventions to be effective,^34^ past research suggests that a higher occurrence of sessions might be more impactful.^52^

Furthermore, the monitoring of biological parameters of participants, like cortisol and blood pressure, as recommended by Ugur et al.,^53^ might confirm or disconfirm the outcomes observed and measured through psychosocial tools.

Also, a follow-up evaluation after at least 12-month post-treatment should have allowed to assess the durability of the SOUND effects and clarify to practitioners the need for a periodic reintroduction of the activities to stabilize initial improvements.

Finally, the quantitative outcomes presented in this paper need to be integrated with qualitative data emerging from observations of the music sessions and the testimonies of older adults and their family caregivers, which will be the topic of another paper.

### Suggestions for full-scale studies

Based on the lesson learned from the SOUND experimentation, full-scale studies, such as RCTs, aimed at demonstrating the effectiveness of music-based rehabilitative interventions, should be cross-national, longitudinal, and involving larger samples. A three-arms-RCT may clarify the effectiveness of individual music interventions versus group music interventions versus no musical/traditional brain stimulation.

Wanting to increase the sample size, the recruitment might not be as difficult as the engagement of participants along the treatment, considering the precarious health conditions of the older population, especially during the winter season, characterized by flu epidemics.

Studies that also include qualitative data, e.g., live and recorded observation of the behavior of OAMD during the music sessions, and collection of participants’ reactions through rapid small interviews, are encouraged, to capture the subtle changes and nuances that sounds and harmonies can trigger. Quantitative data collection tools may be not enough for understanding the impact of art-based interventions. In fact, not everything that is measurable may be important. On the contrary, it may be important to grasp what is meaningful for participants even if not measurable. Music moves certain dimensions—emotional, relational, psychological—that should be observed as a whole in order to be able to grasp the experience lived by people with dementia.

Rigorous testing of the intervention in several countries presupposes that the method is applied homogeneously in every pilot site. This problem was overcome in SOUND with the training of facilitators in the three countries.^42^ However, the socio-economic and long-term care conditions specific to each country can influence the results of the trial, as it was the case in Romania. Therefore, the study protocol should also include measures to control these variables as well.^38^

Moreover, longer experimentations that might ensure a better and longer impact on participants’ well-being, cognition and mood and help identifying possible adverse effects are welcome.

Furthermore, future RCTs are recommended which include the analysis of biological parameters, e.g., cortisol, to identify possible adverse effects of music-based rehabilitative interventions on older people with cognitive decline such as distress leading to anxiety and depression.

A final reflection concerns the participants’ interest in music (that was an inclusion criterion) and how this might bias the impact of the intervention on the studied dimensions. The intervention was received with curiosity and enthusiasm by the DCPs and family caregivers of the OAMD due to its originality and playfulness. During the treatment, OAMD who, according to the biographies compiled at baseline, had always loved music and/or sung or danced in their youth or adulthood, participated in the activities with enthusiasm from the very beginning. However, even the most skeptical and those who had never shown much interest in music, they took pleasure in the activities and became involved with the facilitators after the first two or three meetings. In the three countries, indeed, none of the older adults refused to perform any of the proposed activities nor did they experience sudden deteriorations in their anxiety and depression scales at any particular session. Thus, more than the interest in the musical activities, it was the pleasure the participants derived from them that influenced the participants’ level of engagement and the effects of the intervention of their well-being and mood. It is therefore important that music-based interventions are pleasant, interesting, stimulating, playful and fun to capture the attention and interest of all older participants.

### Conclusions

Despite its piloting nature, the SOUND intervention resulted effective in increasing the levels of well-being, cognitive and executive functions of older adults with mild to moderate dementia. Music-based person-centered interventions should be regularly, not only sporadically, implemented in elder care facilities, as rehabilitative treatments, i.e., with not only recreational purposes.

Such interventions should also be prescribed by medical professionals, e.g., neurologists, along with pharmacological treatments, because they can complement their effectiveness by fostering the fulfilment of psychosocial needs for attachment, comfort, identity, inclusion, occupation and love older adults manifest as the disease progresses.

It is important to provide more evidence on the effectiveness of non-pharmacological music-based interventions in the treatment of dementia, not only to enrich academic knowledge, but also to raise awareness among professionals and managers of care/health units and educational institutions about evidence-based practices that can be replicated in health units, in light of their demonstrated benefits and accessibility.
